# Boronic Derivatives of Thiosemicarbazones as Tyrosinase Inhibitors

**DOI:** 10.3390/pharmaceutics17101300

**Published:** 2025-10-05

**Authors:** Michał Jewgiński, Msanif Msanif, Honorata Zachary, Mateusz Psurski, Rafał Latajka

**Affiliations:** 1 Department of Bioorganic Chemistry, Faculty of Chemistry, Wrocław University of Science and Technology, Wybrzeże Wyspiańskiego 27, 50-370 Wrocław, Poland; msanif.msanif@pwr.edu.pl; 2 Department of Experimental Oncology, Hirszfeld Institute of Immunology and Experimental Therapy, Polish Academy of Sciences, R. Weigla 12, 53-114 Wrocław, Poland; honorata.zachary@hirszfeld.pl (H.Z.); mateusz.psurski@hirszfeld.pl (M.P.)

**Keywords:** inhibitor, molecular modeling, molecular docking, molecular recognition, melanogenesis, structure-activity relationships

## Abstract

**Background:** Tyrosinase is a copper-dependent oxyreductase capable of catalyzing the oxidation of mono- and diphenols. Its activity is crucial in the biosynthetic pathway of melanin, the pigment responsible for the pigmentation of mammalian skin and fur, and protecting their skin from harmful UV radiation. Overproduction of this pigment leads to numerous pathological conditions, including the most severe form of skin cancer—malignant melanoma. Furthermore, tyrosinase produced in plant tissues leads to the browning of damaged vegetables and fruits. Therefore, the search for compounds that effectively and efficiently control tyrosinase activity is desirable for both pharmaceutical and food applications. **Methods**: A group of six boronate derivatives of thiosemicarbazones was synthesized, and their inhibitory properties against tyrosinase were determined. Furthermore, their ability to inhibit melanogenesis and proliferation in SK-MEL-3 and Hs294T cells was investigated. Docking simulations were performed to determine the nature of the inhibitor–protein interactions. **Results**: The tested inhibitors exhibited half-maximal inhibitory concentrations (IC_50_) in the micromolar range. The best inhibitor, compound **6**, had an IC_50_ of 1.4 µM. The tested compounds exhibited poor selectivity for cell lines capable of high and low tyrosinase overexpression, with inhibitor **4** proving to be the most selective compound among those tested. Molecular modeling results indicate that the compounds with the highest activity against tyrosinase interact with the active cavity and the copper ions present within it via a boron moiety substituted on the aromatic ring of the thiosemicarbazones. Cell-based experiments indicated limited antiproliferative effects up to 100 µM across the tested lines. The compounds demonstrated weak antiproliferative effects in SK-MEL-3 and Hs-294T up to 100 µM. **Conclusions**: Our results show that the introduction of a boronic acid moiety is an alternative to carboxylic acid derivatives, improving the inhibitory activity of boron analogs (by fourfold) against fungal tyrosinase.

## 1. Introduction

Tyrosinase (EC 1.14.18.1) is an oxidoreductase with mono- and diphenolase activity, capable of catalyzing two different reactions, respectively, the oxidation of monophenols to o-quinones and the oxidation of o-diphenols to the corresponding o-quinones [1]. Both activities depend on the presence of two cupric ions located in the active center of the enzyme, which are capable of binding an oxygen molecule, the oxidant in the catalyzed chemical reaction [2]. When L-tyrosine or L-dopa are the substrates, the product of the tyrosinase-catalyzed reaction is dopaquinone, an intermediate in the melanin biosynthetic pathway [3].

Melanin is a pigment responsible for the pigmentation of both mammalian skin and fur. It also absorbs UV radiation, protecting the skin from damage and simultaneously removing reactive oxygen species [4]. On the other hand, the overproduction of melanin, which accumulates in the skin, is the cause of numerous pathological conditions, including age spots, melasma, and one of the most severe forms of skin cancer—malignant melanoma [5]. At the same time, it has been found that disturbances in the melanin biosynthesis pathway are associated with some neurodegenerative diseases, such as Parkinson’s, Alzheimer’s, and Huntington’s disease [6].

Tyrosinase, an enzyme widely distributed in plants, is also responsible for undesirable changes from a functional perspective. It is responsible for the enzymatic browning of plant products, leading to the formation of brown or black pigments in both fruits and vegetables. It is one of the primary factors contributing to a decline in their sensory properties, and consequently, a decrease in their functional value [7].

Therefore, the search for compounds that effectively and efficiently control tyrosinase activity is desirable for both pharmaceutical and food applications. Unfortunately, most tyrosinase inhibitors presented in the literature are not suitable for oral or topical use due to their toxicity. Kojic acid and hydroquinone, formerly used as ingredients in skin-lightening cosmetics, have been discontinued due to their toxicity at concentrations that provide a depigmenting effect [8,9].

When searching for potential tyrosinase inhibitors, we can consider compounds capable of coordinating metal ions, especially copper ions, which are present in the active cavity of tyrosinase. Therefore, thiosemicarbazones, representing a versatile class of ligands, constitute an interesting group of compounds. They are characterized by antimalarial [10] and antitumor properties [11,12], and antimicrobial activity [10].

For the last years, thiosemicarbazone derivatives have been used as tyrosinase inhibitors. Compounds having a thiosemicarbazone skeleton with an aromatic ring substituted with -OR groups [11,12,13,14], -NR [15,16], halides [12,17,18,19], or heteroaromatic derivatives [20,21,22,23] are characterized by strong inhibitory properties. These abilities are the result of the structural analogy between the aromatic ring and the natural substrate of tyrosinase, L-tyrosine or L-dopa, which can freely interact with the enzyme’s hydrophobic active cavity. The thiourea moiety is also essential from the point of view of interaction with the enzyme, as it can act as a chelator of catalytically essential copper ions in the active cavity of the enzyme, increasing the coordination abilities of the inhibitor [24,25]

As we showed in our previous work, acetophenone thiosemicarbazones are more potent inhibitors than benzaldehyde thiosemicarbazones. This is the result of a tighter filling and fitting of the acetophenone fragment into the hydrophobic cavity of the enzyme [26].

When examining the literature, it is difficult to find reports on the use of thiosemicarbazone derivatives of boronic acids as tyrosinase inhibitors. Although Hick and colleagues report the synthesis of ortho, meta-, and para-substituted boronic analogues of benzaldehyde thiosemicarbazones, they describe their potential use as antifungal compounds, without considering their ability to inhibit diphenolase activity. Although there are no reports in the literature on the use of boronic acids to inhibit tyrosinase, Belitsky et al. [27] indicate that boronic acids can be used to control melanin polymerization synergistically with tyrosinase inhibitors, or alternatively. It is possible due to the boronic derivative’s ability to form reversible adducts with catechols. Therefore, this work aims to determine the inhibitory capacity of boronate derivatives of thiosemicarbazones against fungal tyrosinase. For this purpose, the activity of the previously described boronate analogues of benzaldehyde, as well as newly synthesized boronate analogues of acetophenone, was determined. This aspect is essential because, as our previous studies have shown, acetophenone derivatives generally have better inhibitory properties than benzaldehyde ones.

**Figure 1:**
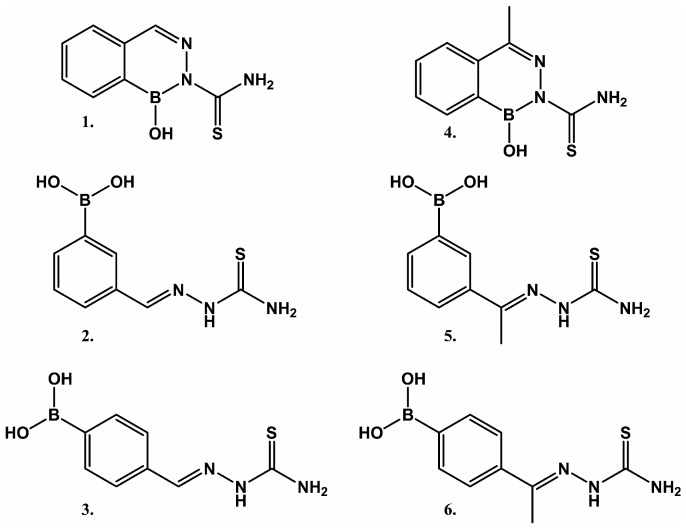
Structures of compounds **1**–**6**.

Furthermore, as Ledwoń and colleagues point out, carboxylate derivatives of benzaldehyde thiosemicarbazones and acetophenone have an IC_50_ value against tyrosinase at the micromolar level. Therefore, considering the similar acidic properties of boronic acids and their superior complexing abilities, an attempt to determine the inhibitory capacity of boronate analogues of thiosemicarbazones seems even more justified. This paper presents the synthesis of all possible boron analogues of benzaldehyde and acetophenone, and their ability to inhibit tyrosinase activity, expressed as IC_50_ values. The efficacy of the tested thiosemicarbazones in inhibiting melanin production was also examined in a panel comprising the SK-MEL-3 melanoma cell line, which exhibits high tyrosinase (TYR) expression, its low-expressing counterpart, Hs294T, and the non-cancerous MCF-10A cell line as a healthy control. The docking process of the tested compounds **1**–**6** (see Figure 1) was also simulated, and their interaction energies with the active cavity of tyrosinase were determined to gain a better understanding of the nature of inhibitor-enzyme interactions. The results of this study may be helpful in the design and development of new tyrosinase inhibitors with potential pharmaceutical and cosmetic applications.

Potent inhibition of mushroom tyrosinase does not always result in clinical or cellular effectiveness. Even when in vitro IC_50_ values are low, cellular responses can be blunted by differences between fungal and human tyrosinase, the ionization state of boronic acids at physiological pH (favoring boronate forms that cross membranes poorly), potential metabolic instability and protein binding, as well as the multifactorial control of melanogenesis. Human tyrosinase, membrane permeability, metabolic stability, and intracellular target engagement were not evaluated in this study; these are still areas that need to be addressed in subsequent research.

## 2. Materials and Methods

### 2.1. Chemistry

General information: The 1H NMR spectra were recorded on a 600 MHz Bruker Avance (Rheinstten, Germany) and a Jeol ECZ 400S (Akishima, Japan) spectrometer in DMSO-d6 as a solvent. Chemical shifts (σ) were expressed in parts per million (ppm) relative to solvent signals (2.54 ppm). High-resolution Mass Spectra were obtained on a Waters Xevo G3 Q-TOF (Milford, CT, USA) apparatus in ESI^+^ mode. All solvents were of commercial quality and purchased from a local supplier (Avantor Performance Materials Poland S.A. (Gliwice, Poland); purity: pure p. a.). Thiosemicarbazide was purchased from Sigma-Aldrich, while the starting aldehydes and acetophenones were from TCI (Haven, Belgium), Fluorochem (Hadfield GB), and Sigma-Aldrich. No procedures or product isolations were optimized.

#### General Procedure for the Synthesis of **1**–**6**

A (formylphenyl)boronic acid or (acetophenone)boronic acid was completely dissolved in 20 mL of hot H_2_O in a 100-mL round-bottomed flask. Next, thiosemicarbazide (in a 1:1 molar ratio) and 5 drops of HCOOH were added. The reaction mixture was refluxed for 2 h. After cooling to room temperature (r.t.), the precipitated product was collected by filtration as a white solid and washed with hexane (3 × 5 mL) and dried in vacuo.

The characteristics of compounds **1**–**3** were already described by Hicks and coworkers [28].

(4) Yield: 89%, ^1^H-NMR (400 MHz, DMSO-d_6_): 10.70 ppm (s, 1H, B(OH)), 9.65 ppm (bs, 1H, -NH_2_), 9.58 ppm (bs, 1H, -NH_2_), 8.12 ppm (d, J = 7.36 Hz, 1H, Har), 7.91 ppm (d, J = 7.94 Hz, 1H, Har), 7.83ppm (t, J = 7.62 Hz, 1H, Har), 7.72ppm (t, J = 7.35 Hz, 1H, Har), 2.59ppm (s, 3H, -CH_3_); ^13^C-NMR (151 MHz, DMSO-d_6_): 185.2 ppm, 146.6 ppm, 134.5 ppm, 133.2 ppm, 132.5 ppm, 130.9 ppm, 126.7 ppm, 20.3 ppm; HRMS (ESI+): *m*/*z* calc for C_9_H_10_BN_3_OS (M+H)^+^ 220.0718, found 220.0724

(5) Yield: 81%, ^1^H-NMR (400MHz, DMSO-d_6_): 10.20 ppm (s, 1H, N = NH), 8.32 ppm (bs, 1H, -NH_2_), 8.25 ppm (s, 1H, Har), 8.11 ppm (s, 2H, B(OH)_2_), 7.95 ppm (d, J = 7.82 Hz, 1H, Har), 7.84ppm (bs, 1H, -NH_2_), 7.74 ppm (d, J = 7.29 Hz, 1H, Har), 7.31 ppm (t, J = 7.58 Hz, 1H, Har), 2.27 ppm (s, 3H, -CH_3_)); ^13^C-NMR (151 MHz, DMSO-d_6_): 179.3 ppm, 148.6 ppm, 137.1 ppm, 135.5 ppm, 132.4 ppm, 128.8 ppm, 128.0 ppm, 14.4 ppm; HRMS (ESI+): *m*/*z* calc for C_9_H_12_BN_3_O_2_S (M+H)^+^ 238.0823, found 238.0822

(6) Yield: 83%, ^1^H-NMR (400MHz, DMSO-d_6_): 10.17 ppm (s, 1H, N = NH), 8.25 ppm (bs, 1H, -NH_2_), 8.06 ppm (s, 2H, B(OH)_2_), 7.90 ppm (bs, 1H, -NH_2_), 7.80 ppm (d, J = 8.06 Hz, 2H, Har), 7.71 ppm (d, J = 8.15 Hz, 2H, Har), 2.25 ppm (s, 3H, -CH_3_); ^13^C-NMR (151 MHz, DMSO-d_6_): 179.2 ppm, 148.4 ppm, 139.5 ppm, 134.5 ppm, 126.0 ppm, 14.4 ppm; HRMS (ESI+): *m*/*z* calc for C_9_H)_12_BN_3_O_2_S (M+H)^+^ 238.0823, found 238.0810

### 2.2. Tyrosinase Enzymatic Assay

Tyrosinase from mushrooms (EC: 1.14.18.1) was purchased from Sigma-Aldrich. The tyrosinase enzymatic assay was performed as described in our previous papers [26,29,30], using L-DOPA (Sigma-Aldrich, St. Louis, MO, USA), 10 mM, dissolved in 0.15 mM orthophosphoric acid solution) as the substrate. All thiosemicarbazones were dissolved in DMSO to concentrations of 50 mM and 100 mM, and then diluted in sodium phosphate buffer (0.1 M, pH 6.8) to the desired test concentrations (DMSO concentrations in reaction mixtures were below 1%).

Tyrosinase solution (50 kU/mL) was diluted 20 times in 0.1 M sodium phosphate buffer (pH 6.8). 10 µL of the diluted enzyme solution was first pre-incubated with the inhibitor solutions for 5 min at 25 °C.

After the L-DOPA solution was added, monitoring of the reaction was initiated by measuring the change in absorbance of the orange-brown product (dopachrome) for 10 min (λ = 475 nm, 25 °C) using a Molecular Devices SpectraMax Plus 384 Microplate Reader (San Jose, CA, USA). The control sample contained the same reagents as the test samples except for the substrate and inhibitor. Kojic acid (Sigma-Aldrich) was used as a positive control and was treated the same way as the other inhibitors. The activity results presented are the average of two measurements.

The optimal ionic strength and pH for tyrosinase activity were provided by a sodium phosphate buffer (0.1 M, pH 6.8).

### 2.3. Molecular Modeling

The protein structure used in the docking process was downloaded from the RSCB PDB database and deposited with 2Y9X [2]. Protein structure was prepared using the Protein Preparation module with simultaneous optimization of hydrogen atom positions. Structures of the studied inhibitors 1–6 were prepared and optimized using the OPLS-2005 force field [31]. The inhibitor structures were docked into the tyrosinase active site using the Induced Fit Docking protocol. For each docking performed with the PosePrediction mode, additional optimization of the obtained complexes was performed within a radius of 5 Å from the docked inhibitor. Additionally, the position of ligands for the three best poses was evaluated using the MMGBSA score. Protein-inhibitor interactions were analyzed using both the Ligand Interaction module of the Schrodinger package 2025-2 (Schrodinger Inc. New York, NY, USA), PyMol version 0.99rc6 (DeLano Scientific LLC, San Carlos, CA, USA), and the Discovery Studio Visualizer v25.1.0.24284 (Dassalut Systemes, San Duego, CA, USA).

### 2.4. Antiproliferative Studies

Reference drugs. Cisplatin was purchased from Ebewe (Unterach am Attersee, Austria) and was maintained as a ready-to-use stock solution (1 mg/mL) at room temperature. Doxorubicin hydrochloride was purchased from Accord Healthcare Polska (Warsaw, Poland) and was stored at +4 °C as a ready-to-use stock solution (2 mg/mL).

Cell lines. The Hs-294T (HTB-140), MCF-10A, and SK-MEL-3 (HTB-69) cell lines were purchased from the American Type Culture Collection (ATCC; Rockville, MD, USA). The KU-19-19 was purchased from Leibniz Institute DSMZ-German Collection of Microorganisms and Cell Cultures GmbH (Braunschweig, Germany). Hs-294T, SK-MEL-3, and KU-19-19 (ACC 395) cell lines were cultured in Dulbecco’s Modified Eagle Medium (DMEM; Biowest, Nuaille, France) supplemented with 10% (*v*/*v*) fetal bovine serum (FBS; GE Healthcare HyClone, Logan, UT, USA), 2 mM L-glutamine (Sigma-Aldrich, Poznań, Poland). The MCF-10A cell line was cultured in Ham’s F12 medium with glutamine (Corning Costar, Warsaw, Poland) supplemented with 5% (*v*/*v*) FBS, 5% (*v*/*v*) horse serum, 10 µg/mL insulin, 0.05 µg/mL cholera toxin, 0.5 µg/mL hydrocortisone, and 20 ng/mL hEGF (all from Sigma-Aldrich). All culture media contained 100 µg/mL streptomycin (Sigma-Aldrich) and 100 U/mL penicillin (PolfaTarchomin SA, Warsaw, Poland). The cell lines were tested for mycoplasma contamination using Venor GeM Classic (Minerva Biolabs, Berlin, Germany) with negative results in all cases. Cell lines were cultured in a humidified atmosphere at 37 °C with 5% (*v*/*v*) CO_2_ and passaged twice a week using ethylenediaminetetraacetic acid (EDTA)-Trypsin solution (pH 8; HIIET, Wroclaw, Poland) as a detachment agent.

Antiproliferative activity assessment by sulforhodamine B assay. The cells were seeded on 384-well plates (Greiner Bio One, Kremsmünster, Austria) at a density of 1 × 10^3^ cells/well and, after overnight attachment, were exposed to compounds at various concentrations (at least eight concentrations). After 72 h, the sulforhodamine B (SRB) assay based on Skehan et al. [32] was performed with minor modifications. Briefly, cell were fixed with 20% (*w*/*v*) trichloroacetic acid (Avantor Performance Materials, Gliwice, Poland) for 1 h, washed several times with tap water, and stained with 20 µL of 0.1% (*w*/*v*) solution of sulforhodamine B (Sigma-Aldrich, Poznan, Poland) in 1% (*v*/*v*) acetic acid (Avantor Performance Materials, Gliwice, Poland). After 30 min of incubation at room temperature, the unbound dye was washed out with 1% (*v*/*v*) acetic acid, whereas the bound dye was solubilized with 70 µL of a 10 mM unbuffered tris(hydroxymethyl)aminomethane (TRIS; Avantor Performance Materials, Gliwice, Poland) solution. The entire procedure was performed using Biotek EL-406 washing station (BioTek Instruments, Winooski, VT, USA). Absorbance was read using a Biotek Hybrid H4 reader (BioTek Instruments, Winooski, VT, USA) at 540 nm wavelength. Proliferation inhibition (%Inh) was calculated using the following formula: %Inh = [((A_p − A_m)/(A_k − A_m)) × 100] − 100, where Am—absorbance of cell-free medium (blank), Ak—absorbance for vehicle-treated cells (control), Ap—absorbance for compound-treated cells. IC_50_ (95% CI) values were calculated in GraphPad Prism v. 10.5.0 (GraphPad Software Inc., San Diego, CA, USA) using the [inhibitor] vs. response—variable slope (four parameters) Hill’s equation, based on at least three biological, independent repeats. The results are presented as IC_50_ ± 95% confidence intervals.

## 3. Results and Discussion

### 3.1. Chemistry

**Scheme 1:**
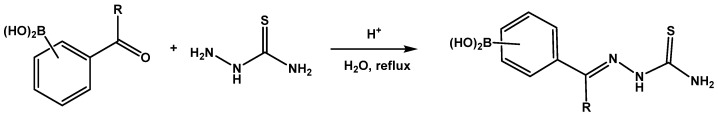
General procedure for the synthesis of investigated thiosemicarbazones.

The structure of all investigated compounds is shown in Figure 1. All compounds were synthesized via the condensation of carbonyl compounds with thiosemicarbazide using a catalytic amount of acetic acid in a boiling water solution (see Scheme 1). After 2 h of reflux and cooling to room temperature, the desired compounds precipitate and are then washed with hexane, yielding the compounds in good to excellent yields. Compounds **1**–**3** have been previously described by Hick et al. [1]; compounds **4**–**6** have not been reported before. All obtained compounds have been characterized by ^1^H and ^13^C NMR spectroscopy, as well as high-resolution mass spectrometry. Spectra, mainly those newly obtained, are available in the Supplementary Materials.

Analysis of the ^1^H NMR spectra for all obtained compounds shows that, for all of them, a signal from the amide proton of the =N-NH- moiety is observed at a chemical shift higher than 10 ppm. Such a chemical shift value is recognized only when the thiosemicarbazone adopts the *E* isomer. In case of *Z* isomer, the signal for this proton would be found at a magnetic field at a chemical shift in the range 8.5–9 ppm [29]. Based on this information and 2D NOESY spectra, it can be concluded that the investigated thiosemicarbazones exist as the E isomer. This type of isomer was considered in further studies, including molecular modeling. A similar tendency has been reported for the other analogs of thiosemicarbazones investigated in our group [19,26,29].

### 3.2. Effect of Compound ***1***–***6*** on the Diphenolase Activity of Mushroom Tyrosinase

The high structural and functional homology between *Agaricus bisporus* tyrosinase and mammalian tyrosinase is why fungal tyrosinase is most commonly used in enzymatic assays. The readily commercial availability and convenient enzymatic assay undoubtedly contribute to the widespread use of Agaricus bisporus tyrosinase [33,34,35]. Therefore, during the activity tests of the tested compounds, fungal tyrosinase was used.

All compounds were tested at various concentrations, from 0.2 μM to 4 mM, after incubating with tyrosinase in solution for 5 min at room temperature. After this period, the substrate L-DOPA was added, and the enzymatic reaction was immediately monitored. The reaction progress was tracked by measuring the absorbance of the colored product, dopachrome, formed during the reaction at 5-s intervals over 10 min. The percentage tyrosinase activity for each concentration was calculated as the ratio of the linear slope of the absorbance-time graph for the reaction with the inhibitor to that of the control reaction without the inhibitor.

**Figure 2:**
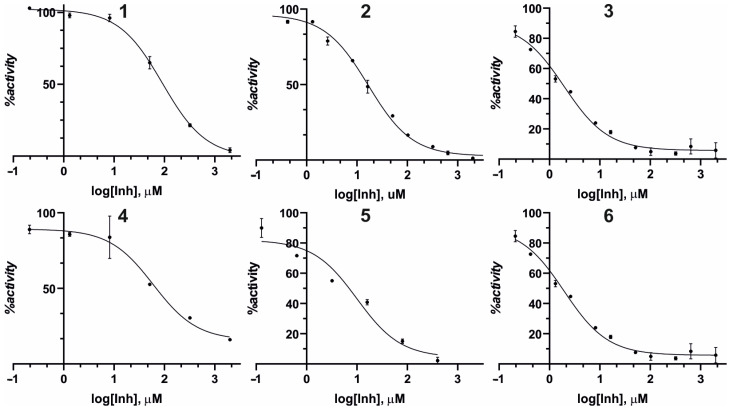
Dose–response curves for the determination of IC_50_ for **1**–**6** and the control compound kojic acid (**KA**) towards mushroom tyrosinase. The concentration of L-DOPA was 1 mM in all cases. Inhibitor concentration ranged from 0.2 μM to 4 mM.

The effect of compounds **1**–**6** and kojic acid, used as a positive control, on enzyme diphenolase activity was expressed as IC_50_, which defines the inhibitor concentration required to inhibit activity by half. For this purpose, a dose–response analysis was performed. A dose–response relationship was investigated by creating curves representing the percentage of relative activity as a function of the inhibitor concentration. Dose–response curves for all investigated compounds are shown in Figure 2. As demonstrated in the figure, all investigated compounds showed a dose-dependent manner of inhibition. The dose–response curves enable the determination of IC_50_ values, which are listed in Table 1. Calculation of the IC_50_ values was performed using GraphPad Prism software [36]. All investigated inhibitors demonstrate the IC_50_ values in the micromolar range.

### 3.3. Structure-Activity Relationship

As indicated by the obtained results, the inhibitory activity of thiosemicarbazones derived from both ortho-borono-benzaldehyde and ortho-borono-acetophenone is lower than that of kojic acid, reaching 87.9 µM and 66.1 µM, respectively. For the remaining analogues, which contain a boronic group in the meta and para positions, the inhibitory activity is comparable to that of **KA**. The obtained IC_50_ values enable us to conclude that acetophenone analogues exhibit an order of magnitude better activity compared to benzaldehyde analogues. This trend is consistent with our previous results on halogen derivatives of benzaldehyde and acetophenone. As shown by Hałdys et al., regardless of the halogen atom introduced in the meta and para positions of the benzyl substituent, acetophenone analogues exhibited IC_50_ values at least an order of magnitude lower than those of benzaldehyde analogues [19]. The only exceptions were analogues of ortho-substituted halogen derivatives, for which the activities of the benzaldehyde homologues were significantly better than those of the acetophenone homologues. However, in the case of **1** and **4**, this trend appears to be slightly reversed, with **4**, an acetophenone derivative, showing a somewhat lower IC_50_ value of 66.1 µM compared to 87.9 μM for **1**. This is most likely because 1 and 4 undergo internal cyclization during condensation, and the additional methyl group present in 4 provides additional intermolecular interactions by filling the hydrophobic cavity. However, a comparison of the effects of the boron group in the meta and para positions indicates that the para-substituted derivatives exhibit better affinity for tyrosinase.

Furthermore, this tendency is stronger for the acetophenone analogues than for the benzaldehyde analogues. This tendency is reflected by the IC_50_ values of 16.3 µM, 11.7 µM, 9.9 µM, and 1.4 µM for **2**, **3**, **5**, and **6**, respectively. Furthermore, a comparison of the effect on the inhibition capacity of boronic acid derivatives and their carboxylic acid derivatives indicates that in the case of the para-substituted analogues, the IC_50_ value for **6** is four times lower than for the corresponding carboxylic acid [30].

### 3.4. Molecular Docking

Understanding the nature of inhibitor-host interactions is a crucial element in the rational design of new inhibitor groups, facilitating the development of novel classes of biologically active compounds. Computational techniques are one method for understanding this nature of interactions, and in this case, methods that simulate the docking process of ligands to the host surface are beneficial. This method enables us to determine the nature and type of intermolecular interactions, thereby understanding how inhibitors interact with the protein surface. The structures of the studied inhibitors **1**–**6** were optimized using the OPLS-2005 force field, preserving the E isomerism of the thiosemicarbazone binding site, as determined by experimental data. The structure of tyrosinase was retrieved from the PDB database, where it is deposited under the code 2Y9X. Subsequently, after protonation, considering the pH used in enzymatic studies (6.8), the docking site was determined based on the tropolone molecule contained in the retrieved enzyme structure. In the next step, the docking process was simulated using the Induced Fit Docking protocol, yielding three poses with the best IFD score for each inhibitor (see Figure S5 and Table 2). For each of these, the inhibitor–protein interaction energy was determined using the MM-GBSA algorithm implemented in Schrodinger 2025-2 software. At this stage of the calculations, the VSGB solvation model was employed, and the positions of all protein residues within a 3 Å radius of the ligand molecule were optimized. The obtained ligand-protein interaction energies are presented in Table 2. The binding poses mirror the SAR. Analogs of para-boronic acid have a higher potency because they align the boronate with the dicopper active site. By contrast, bicyclic analogues **1** and **4** adopt thiourea-oriented poses, in line with their lower activity.

Analyzing the obtained docking results, particularly the information on the inhibitor–protein interaction energy, we can conclude that the lowest interaction energy was observed for bicyclic inhibitors **1** and **4**. These energies are lower by an average of 10 kcal/mol compared to the other inhibitors. These results are consistent with the results of experimental studies, in which inhibitors **1** and **4** exhibited the poorest inhibitory properties against tyrosinase.

**Figure 3:**
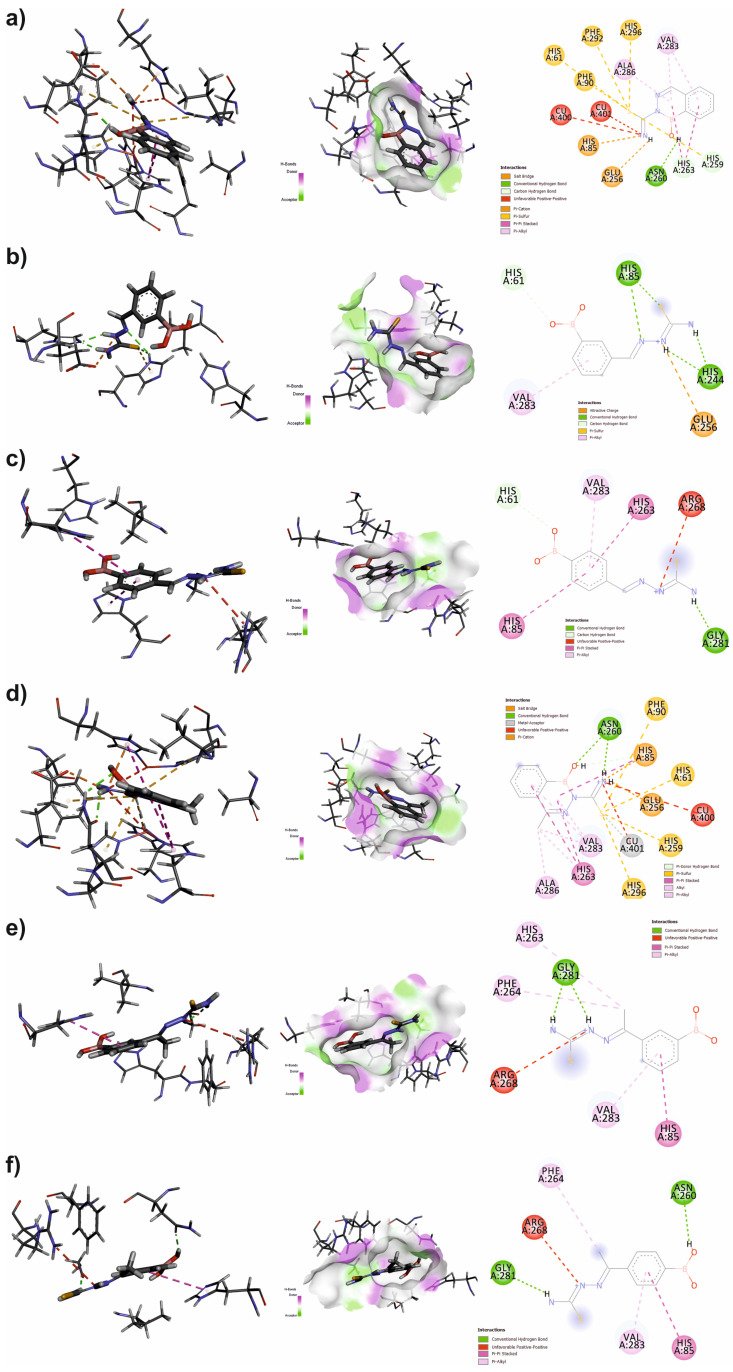
Arrangement of inhibitors (**a**) **1**; (**b**) **2**; (**c**) **3**; (**d**) **4**; (**e**) **5**; (**f**) **6** in the active cavity of tyrosinase characterized by the MM-GBSA lowest-energy. Left column visualization of the inhibitor–protein interactions shown: H-bond—green, pi-pi stacking—pink, charge-charge—red, salt bridges—orange, pi-sulfur—yellow; middle column visualization of the tyrosinase surface with H-bond donor and acceptor sites marked; right column 2D representation of protein-inhibitor interactions.

Furthermore, a closer examination of the arrangement of both inhibitors **1** and **4** with the other inhibitors reveals obvious differences in their orientation within the active cavity. In both compounds, it is the thiourea moiety that is located within the active cavity, and not the boronic acid moiety, as in the other compounds (see Figure 3). The distance of the sulfur trop from the copper ions in both cases is between 2.7 and 2.9 Å (see Figure S8). Due to the orientation of inhibitors **1** and **4**, the hydrophobic ring is exposed outside the hydrophobic active cavity, facing the hydrophilic medium. This arrangement may be responsible for the reduced intermolecular interaction energy.

However, in the case of the remaining inhibitors, **2**, **3**, **5**, and **6**, the aromatic ring with the boronic acid group is the fragment that penetrates the active cavity (see Figure 3), and the distances between the oxygens of the boronic group and the cupric ions range from 2.3 to 4.5 Å. This orientation of the inhibitors, where the thiourea moiety is exposed outside the active cavity, differs from that previously reported for halogenated derivatives of thiosemicarbazones [29]. As a result of docking simulations, we postulated that the inhibitors interacted with the tyrosinase active cavity via the thiourea moiety. However, when studying analogues containing a carboxylic acid moiety in the aromatic ring, it is precisely this acid group that interacts with the active cavity and the cupric ions contained therein [30]. Therefore, considering the similar nature of the boronic and carboxylic acid moieties, the obtained modeling results for inhibitors **2**, **3**, **5**, and **6** confirm the previously observed trend.

Comparing the interaction energy values for inhibitors **2**, **3**, **5**, and **6** reveals no significant trend in their interaction strength with the tyrosinase surface, as indicated by the analysis of IC_50_ values. This observation may be due to the relatively similar mode of ligand-protein interaction and the fact that while local optimization of the complex geometry was allowed during the calculation of the interaction energy (up to 3 Ang from the inhibitor location), a full or significantly extended optimization of the complex geometry might be necessary.

### 3.5. Cytotoxicity

In view of the results obtained from studies on the inhibitory activity of boronate derivatives of thiosemicarbazones against tyrosinase, we decided to test their ability to inhibit the growth and proliferation of tumor lines that overexpress tyrosinase. For this purpose, we tested antiproliferative activity on selected cell lines. Initial antiproliferative screening was conducted using a tissue-agnostic approach on a select panel of cell lines. These lines were selected based on their tyrosinase expression levels and overall dissimilarity, as revealed by a Uniform Manifold Approximation and Projection (UMAP) analysis of the Cancer Cell Line Encyclopedia (CCLE) (see Figure S4). The panel included SK-MEL-3, a melanoma cell line with high tyrosinase (TYR) expression, and its low-expression counterpart, Hs294T. Additionally, the KU-19-19 cell line was chosen for its distinct clustering in the UMAP analysis, while the non-cancerous MCF-10A line served as a healthy control.

**Figure 4:**
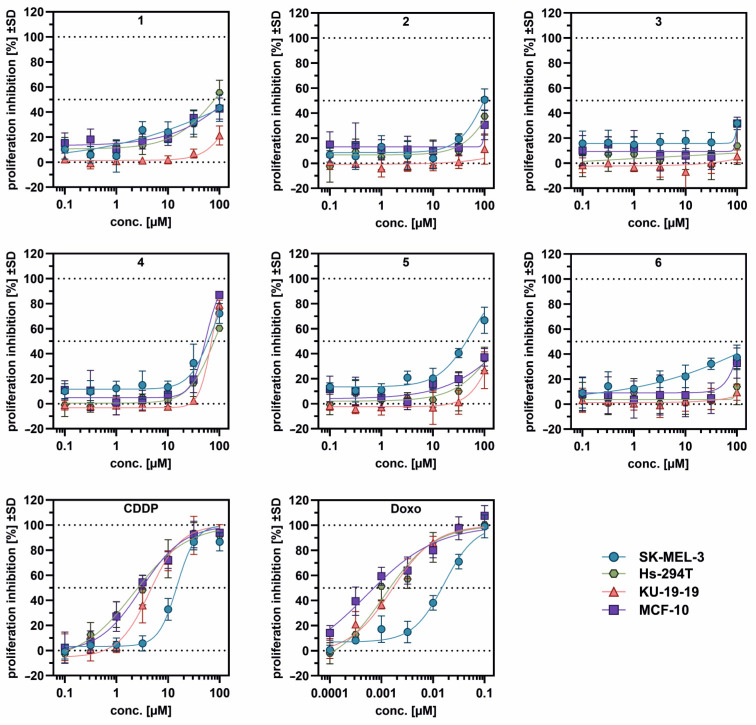
Dose–response curves for the evaluated compounds. Data points represent the mean ± standard deviation (SD) of three independent experiments. The solid lines are nonlinear regression curves generated by fitting the data to the Hill equation.

The majority of the evaluated compounds demonstrated minimal antiproliferative effects up to the maximum tested concentration of 100 µM. All detailed results are presented in Figure 4 and Table 3. Compound **4** was the sole exception, displaying IC_50_ values in the range of 55.0 to 79.4 µM; however, it did not exhibit discernible cell line selectivity. Due to the weak activity of 4 in enzymatic assays (IC_50_: 66.1 µM) and considering that this compound does not show selectivity towards investigated cell lines with high tyrosinase production, its antiproliferative properties most likely have a mechanism unrelated to the selected molecular target—tyrosinase. In contrast, compound **5** showed modest selectivity for the TYR-high SK-MEL-3 cell line, with a moderate IC_50_ value of 56.1 µM.

Additionally, considering that **5** is the second most active compound among those tested, it may indicate that it could be a promising lead compound to obtain a compound characterized by both good tyrosinase inhibiting properties and good antiproliferative properties towards a melanoma cell line with high tyrosinase expression. It is noteworthy that both compounds **3** and **6**, which exhibit the highest tyrosinase inhibitory properties in their groups (IC_50_ values of 11.7 µM and 1.4 µM, respectively), exhibit minimal antiproliferative effects up to a concentration of 100 µM. This observation seems to confirm the fact noted for **4** that the antiproliferative activity is associated with a mechanism other than tyrosinase inhibition. It is also noteworthy that the SK-MEL-3 cell line displayed significant resistance to the standard chemotherapeutics cisplatin (CDDP) and doxorubicin, with IC_50_ values for these agents being 6.9-fold and 14.5-fold higher, respectively, compared to the Hs294T cell line, whereas **4** was equally active on both cell lines, indicating it as potentially not susceptible to common drug-resistance mechanisms.

The observed discrepancy between fungal tyrosinase inhibitory activity and the ability to inhibit proliferation in cancer cells with high and low expression of this enzyme may be due to at least two reasons. The first is that the model fungal tyrosinase used in the in vitro tests and human tyrosinase share only 23% sequence similarity. As Oyama and colleagues [37] point out, this may result in different interactions with hydroxyl-rich stilbene derivatives. The second reason may be due to the limited bioavailability of the tested inhibitors. Observed phenomena may be due to the limited ability of the tested compounds to penetrate the cell membrane. The first, obvious solution to this limitation is to increase the lipophilic nature of these compounds. Unfortunately, attempting to modify the aromatic ring with a lipophilic substituent will significantly increase steric hindrance, preventing adequate penetration of the enzyme’s active cavity. However, introducing a lipophilic moiety onto the free anino group of the thiourea moiety may lead to a complete loss of the compounds’ inhibitory properties, because, as our previous studies have shown, the free amino group of the thiourea moiety is necessary for the tyrosinase-inhibiting activity of thiosemicarbazones [26]. A solution worth considering in further research is encapsulation of the tested inhibitors and their delivery to cells in this form. As the results already published show, this method can be used to distribute biologically active compounds effectively [38,39,40].

## 4. Conclusions

In this study, boronate derivatives of benzaldehyde and acetophenone thiosemicarbazone were synthesized, and their inhibitory activity against fungal tyrosinase was assessed. Additionally, molecular docking of these compounds to the enzyme’s active site was conducted, and their ability to inhibit melanogenesis and cell proliferation in melanoma cell lines with high tyrosinase (TYR) expression—SK-MEL-3—and its low-expression counterpart, Hs294T, was investigated.

Dose–response analysis revealed that all tested thiosemicarbazones inhibit tyrosinase diphenolase activity at the micromolar level. Analyzing the obtained results and considering the dependence of biological activity on the structure of the inhibitors, several general conclusions can be drawn. First, acetophenone derivatives **4**, **5**, and **6** exhibit better inhibitory properties than benzaldehyde derivatives **1**, **2**, and **3**. These conclusions are consistent with our previous studies [19,29]. Furthermore, there is an apparent effect of boronic acid substitution at the ortho, meta, and para positions on the activity of individual analogues. Ortho-substituted derivatives exhibit the lowest activity, while para-substituted derivatives exhibit the highest activity. The differences in the interactions between meta- and para-substituted derivatives can be explained by the results of docking simulations. A detailed analysis of the positions of inhibitors **2**, **3**, **5**, and **6** in the active cavity of tyrosinase indicates a specific trend. In both cases **2** and **5**, two unfavorable interactions between the inhibitor and histidine rings 85 and 263, respectively, can be observed. This unfavorable interaction results from the interaction of the lone electron pairs of both oxygens of the boronate moiety in case **2** and one oxygen of the boronate moiety in case **5** with the NE2 nitrogens of the histidines. In the case of para-substituted analogs **3** and **6**, such an unfavorable interaction does not occur, but an intermolecular hydrogen bond can be observed between the boronate group and the carbonyl oxygen of the Asn260 side chain of tyrosinase. Cytotoxicity tests conducted on melanoma cell lines capable of high tyrosinase overproduction (SK-MEL-3) and low tyrosinase overproduction (Hs294T) indicate that the tested compounds exhibit minimal antiproliferative effects. Compound **4** has the best IC_50_ value, but it did not demonstrate any discernible selectivity for these cell lines. Among the inhibitors tested, inhibitor **5** showed the best selectivity for the SK-MEL-3 cell line with high tyrosinase levels and a moderate IC_50_ value (56.1 µM).

Compound **4** was the only exception, exhibiting IC_50_ values ranging from 55.0 to 79.4 µM; however, its activity mode seems not to be related to tyrosinase as a molecular target.

Our results show that the introduction of a boronic acid moiety is an alternative to carboxylic acid derivatives, improving the inhibitory activity of boron analogs (by fourfold) against fungal tyrosinase. The obtained research results encourage further exploration of the topic. Further research should investigate the inconsistent effects of the tested compounds on enzymatic activity inhibition and their ability to inhibit the proliferation of cancer cells with low and high tyrosinase production in vitro. This aspect is critical in the context of the medical applications of the proposed tyrosinase inhibitors.

## Figures and Tables

**Table 1:** IC_50_ values of the studied compounds on the diphenolase activity of mushroom tyrosinase.

Inhibitor	IC_50_ ^a^ [μM]	95% CI ^b^ IC_50_ [µM]
1	87.9	70.6–112.5
2	16.3	13.6–19.4
3	11.7	9.8–13.9
4	66.1	51.1–91.3
5	9.9	3.2–28.5
6	1.4	0.6–2.3
KA ^c^	14.3	10.9–18.4

^a^ Inhibitor concentration which is required for 50% inhibition, ^b^ confidence interval, ^c^ Dose–response curves for KA see.

**Table 2:** Results of InducedFit docking of the investigated inhibitors.

Inhibitor		IFD Score	MMGBSA dG Binding Energy [kcal/mol]
1	pose 1	−807.44	−22.91
	pose 2	−807.33	−22.69
	pose 3	−807.28	−23.15
2	pose 1	−809.80	**−35.32**
	pose 2	−809.11	−28.33
	pose 3	−809.03	−25.21
3	pose 1	−808.78	−28.20
	pose 2	−808,47	−28.32
	pose 3	−808.08	−34.63
4	pose 1	−810.81	−27.99
	pose 2	−810.50	−24.57
	pose 3	−810.06	−25.00
5	pose 1	−809.74	−35.36
	pose 2	−808.00	−26.44
	pose 3	−806.93	−35.58
6	pose 1	−809.98	−35.13
	pose 2	−809.25	−33.48
	pose 3	−808.88	−31.93

**Table 3:** Summary of the antiproliferative activity of the tested compounds, with cisplatin (CDDP) and doxorubicin included as reference agents.

	IC_50_ [µM] ± 95%CI *							
	SK-MEL-3		Hs-294T		KU-19-19		MCF-10A	
**1**	[*43.4*] *	29.9–56.8	[*55.4*] *	43–67.8	[*21.4*] *	11.9–30.8	[*42.7*] *	34.9–50.6
**2**	[*50.7*] *	39.9–61.6	[*37.6*] *	18.8–56.4	[*11.3*] *	−1.4–24	[*30.7*] *	15.9–45.5
**3**	[*31.8*] *	29.1–34.6	[*13.9*] *	−4.5–32.2	[*5.4*] *	−1–11.7	[*31.7*] *	25.4–38
**4**	**68.5**	**57.8–80.5**	**79.4**	**65.3–97.5**	**69.0**	**64.5–74.4**	**55.0**	**43.2–70.8**
**5**	**56.1**	**48.9–64.3**	[*35.9*] *	26.1–45.7	[*26.8*] *	13.2–40.5	[*37.1*] *	29.7–44.5
**6**	[*37.3*] *	28.2–46.4	[*13.9*] *	−4–31.9	[*9.5*] *	−0.8–19.8	[*32.8*] *	13.6–52
**CDDP**	**15**	**13.7–16.4**	**2.17**	**1.15–3.30**	**4.46**	**3.86–5.14**	**3.03**	**2.40–3.71**
**Doxo**	**0.016**	**0.013–0.021**	**0.0011**	**0.00058–0.0017**	**0.0016**	**0.0011–0.0022**	**0.00051**	**0.0010–0.0023**

*—if IC_50_ > 100 µM; proliferation inhibition at 100µM is reported ± 95% CI as [Mean] ± 95% CI marked with italic.

## Data Availability

The raw data supporting the conclusions of this article will be made available by the authors on request.

## Supplementary Materials

The following supporting information can be downloaded at: https://www.mdpi.com/article/10.3390/pharmaceutics17101300/s1, Figure S1: Spectra for compound **4** (a) 1H NMR, (b) 13C NMR, (c) HRMS; Figure S2: Spectra for compound **5** (a) 1H NMR, (b) 13C NMR, (c) HRMS; Figure S3: Spectra for compound **6** (a) 1H NMR, (b) 13C NMR, (c) HRMS; Figure S4: Two-dimensional NOESY spectra for compound **3**; Figure S5: Dose–response curves for the determination of IC_50_ for kojic acid (KA); Figure S6: Visualization of the Cancer Cell Line Encyclopedia (CCLE) using Uniform Manifold Approximation and Projection (UMAP). The plot highlights the cell lines chosen for this work, and the color scale corresponds to their respective tyrosinase (TYR) gene expression levels. The analysis was performed using the R2 online platform (r2.amc.nl).; Figure S7: Visualization of alignment of the three best poses for (a) **1**; (b) **2**; (c) **3**; (d) **4**; (e) **5**; (f) **6** in the tyrosine active site. Inhibitors are shown as blue sticks, enzyme residues are shown as green lines, and copper ions are shown as gold spheres. Enzyme’s residues involved in the intermolecular H-bond are labeled, and H-bonds are shown as a dashed line.; Figure S8: Distances of the thiourea sulfur atom for inhibitors 1 (top) and 4 (bottom) from the cupric ions of tyrosinase. Cupric ions are marked as gold spheres.; Figure S9: Distances of oxygen atoms of the boron group for inhibitors (a) 2; (b) 3; (c) 5, (d) 6 from the cupric ions of tyrosinase. Cupric ions marked as gold spheres; Figure S10: Alignment of residues 2, 3, 5, and 6 in the tyrosinase active site, with marker unfavorable interactions (orange dashed line) between the boronic oxygen and the atom NE2 of the histidine sidechain ring. The yellow dashed lines represent intermolecular hydrogen bonds.

## Abbreviations

The following abbreviations are used in this manuscript:

KA	Kojic Acid
