# Supplementary material for: Boronic Derivatives of Thiosemicarbazones as Tyrosinase Inhibitors
Source: Pharmaceutics. 2025 Oct 5;17(10):1300. doi: 10.3390/pharmaceutics17101300 (PMC12566723; doi:10.3390/pharmaceutics17101300)
Supplement: Supplementary file 1 [file pharmaceutics-17-01300-s001.zip › pharmaceutics-3857628-supplementary.pdf]

Boronic Derivatives of Thiosemicarbazones as Tyrosinase Inhibitors

Michał Jewgiński [1,*], Msanif Msanif [1], Honorata Zachary [2], Mateusz Psurski [2] and Rafał Latajka [1,*]

[1] Department of Bioorganic Chemistry, Faculty of Chemistry, Wrocław University of Science and Technology, Wybrzeże Wyspiańskiego 27, 50-370 Wrocław, Poland; msanif.msanif@pwr.edu.pl

[2] Department of Experimental Oncology, Hirszfeld Institute of Immunology and Experimental Therapy, Polish Academy of Sciences, R. Weigla 12, 53-114 Wrocław, Poland; honorata.zachary@hirszfeld.pl (H.Z.); mateusz.psurski@hirszfeld.pl (M.P.)

* Correspondence: michal.jewginski@pwr.edu.pl (M.J.); rafal.latajka@pwr.edu.pl (R.L.); Tel.: +48-71-320-24-61 (M.J.); +48-71-3202463 (R.L.)

a

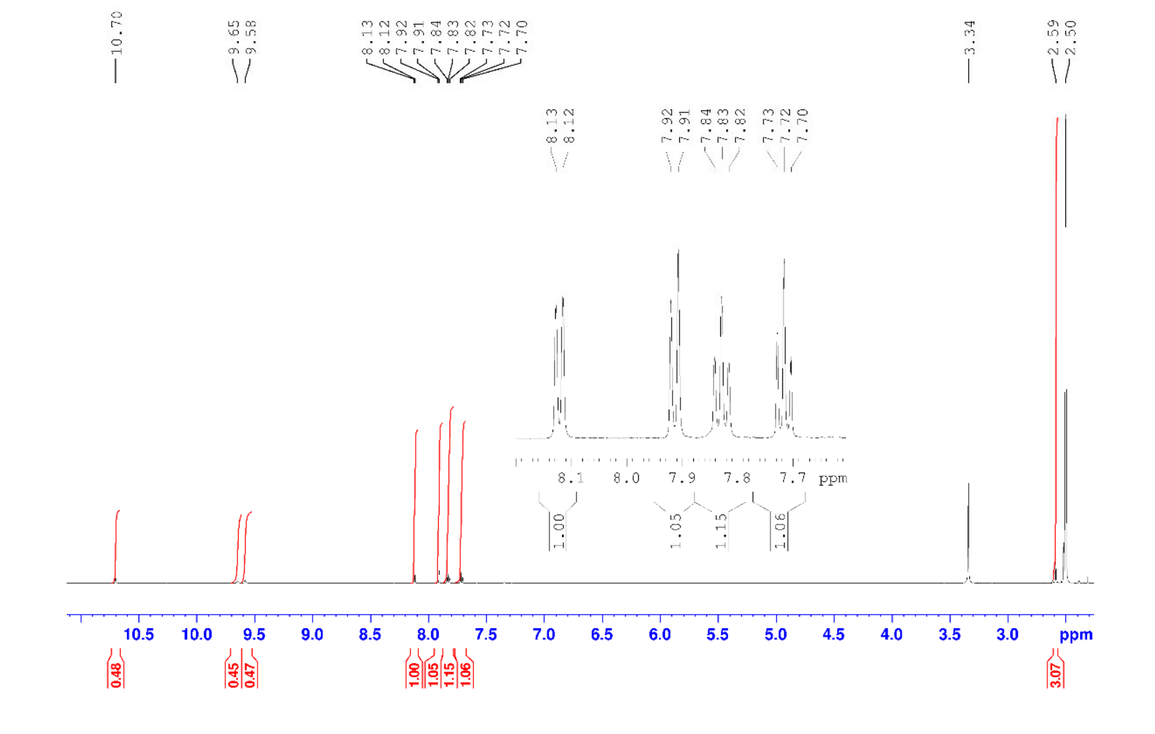

b

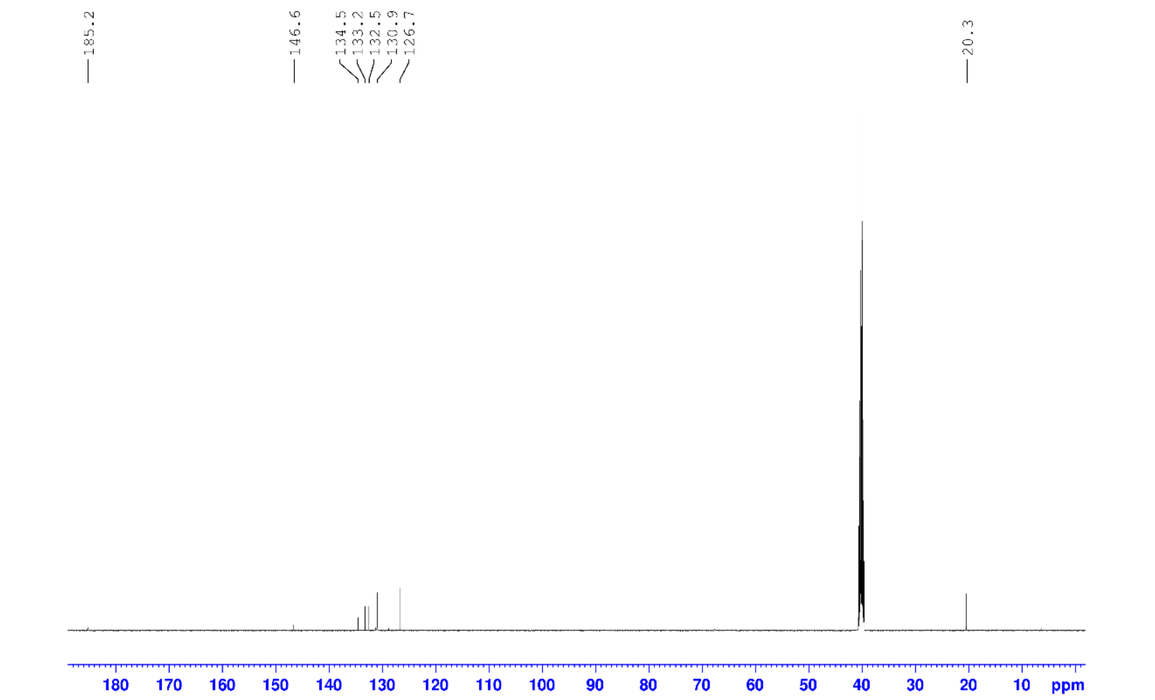

c

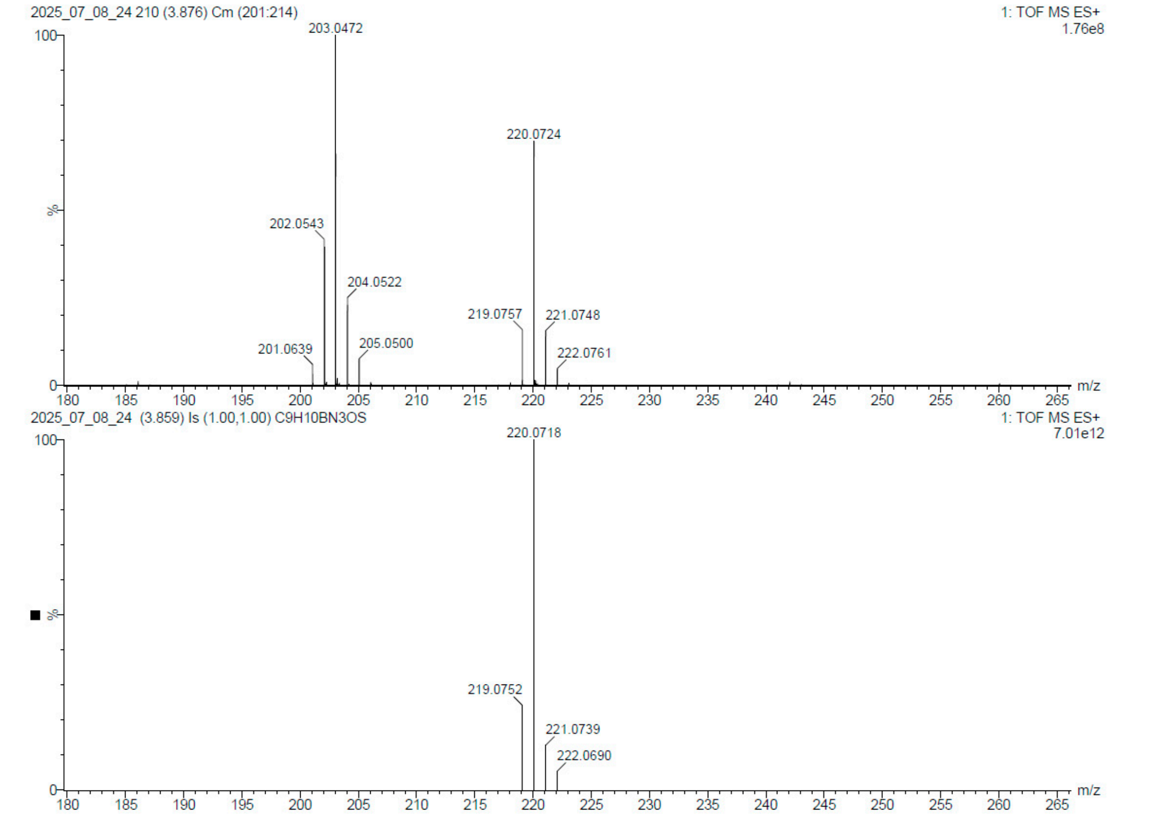

Figure S1. Spectra for compound **4** a) ^{1}H NMR, b) ^{13}C NMR, c) HRMS

a

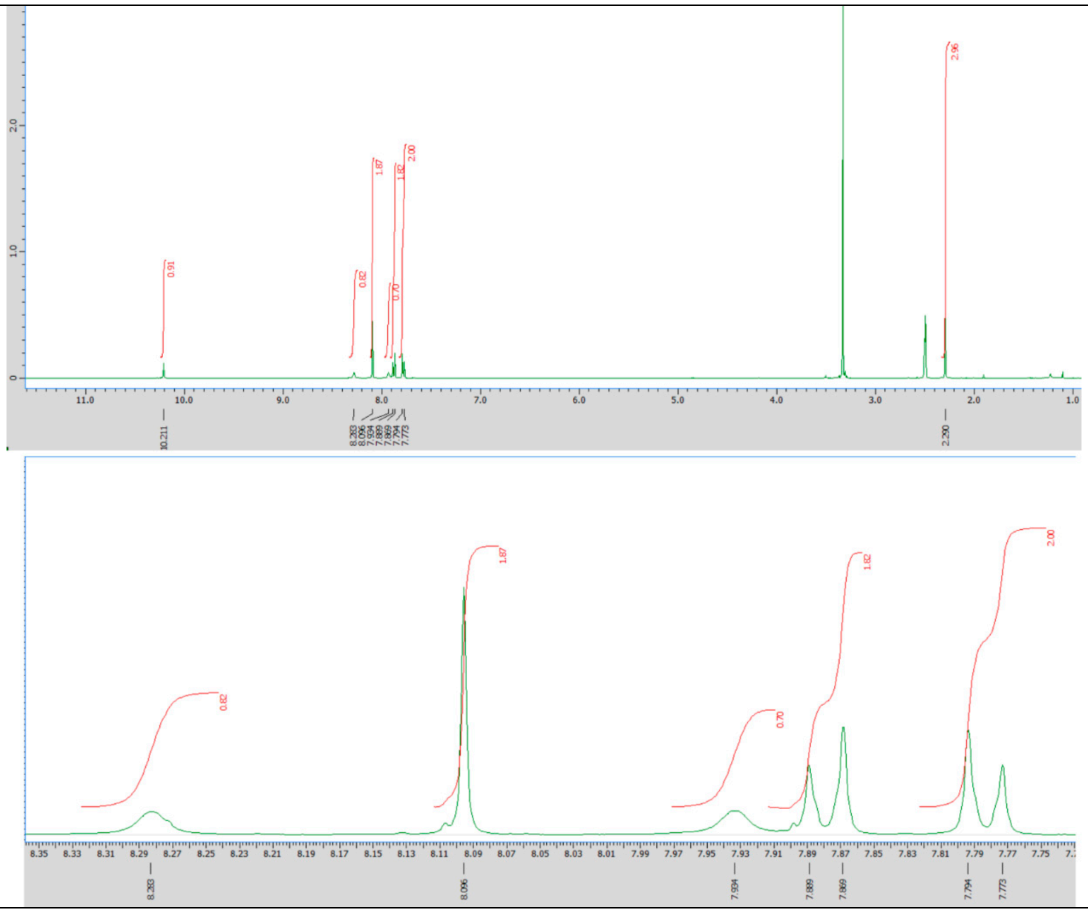

b

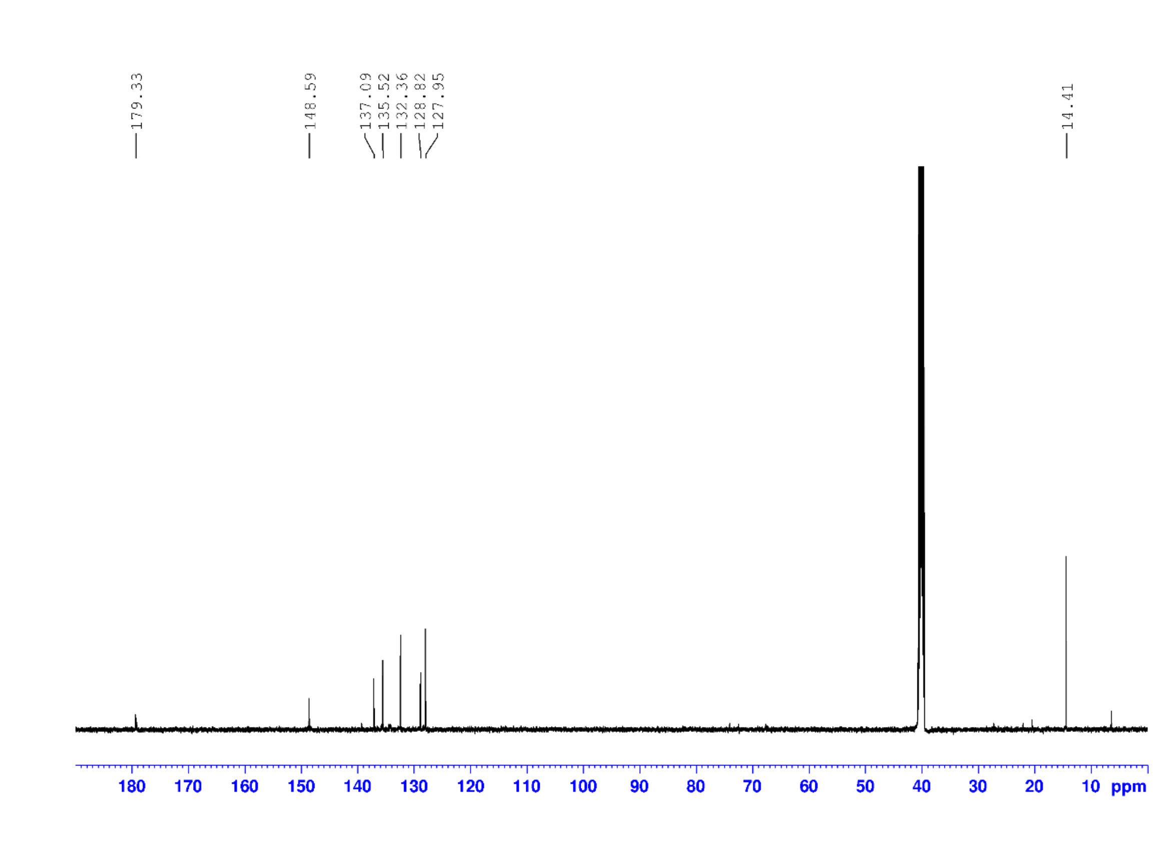

c

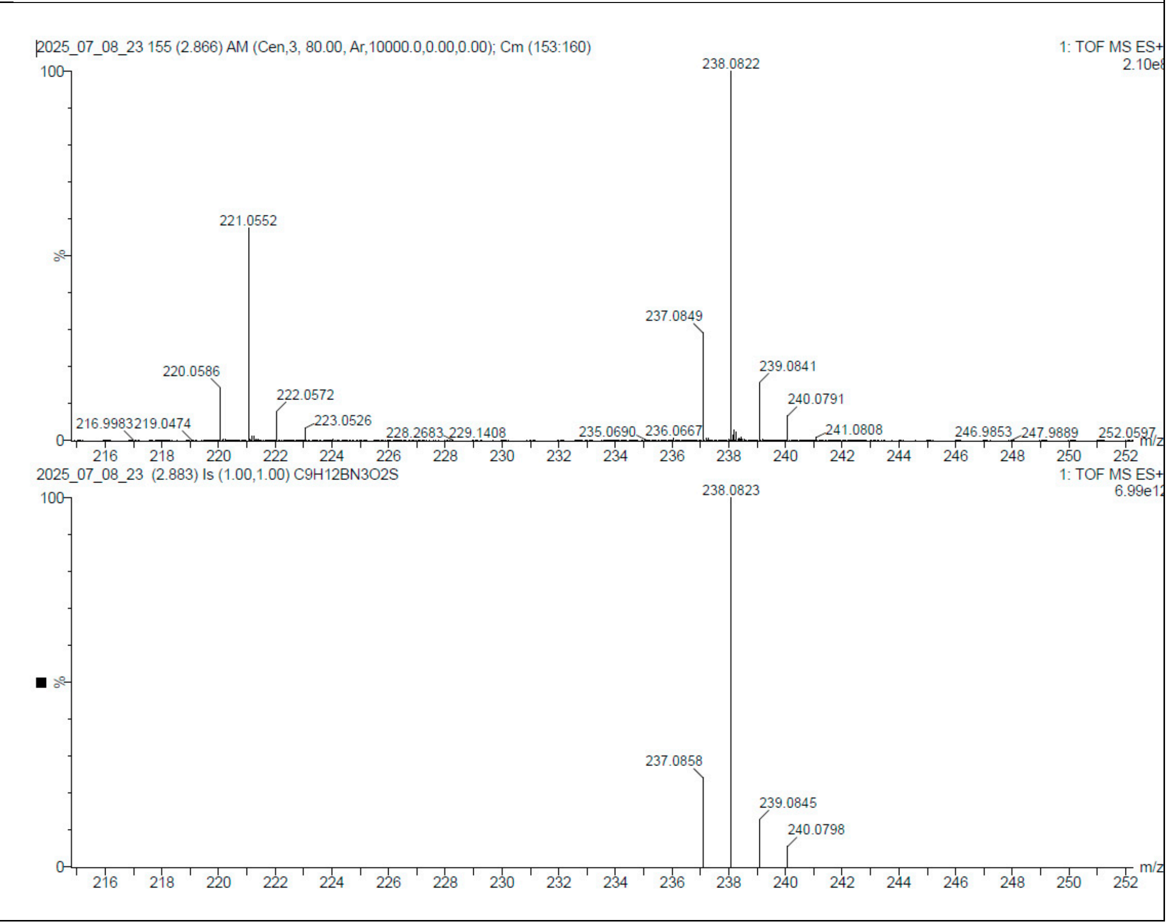

Figure S2. Spectra for compound **5** a) ^{1}H NMR, b) ^{13}C NMR, c) HRMS

a

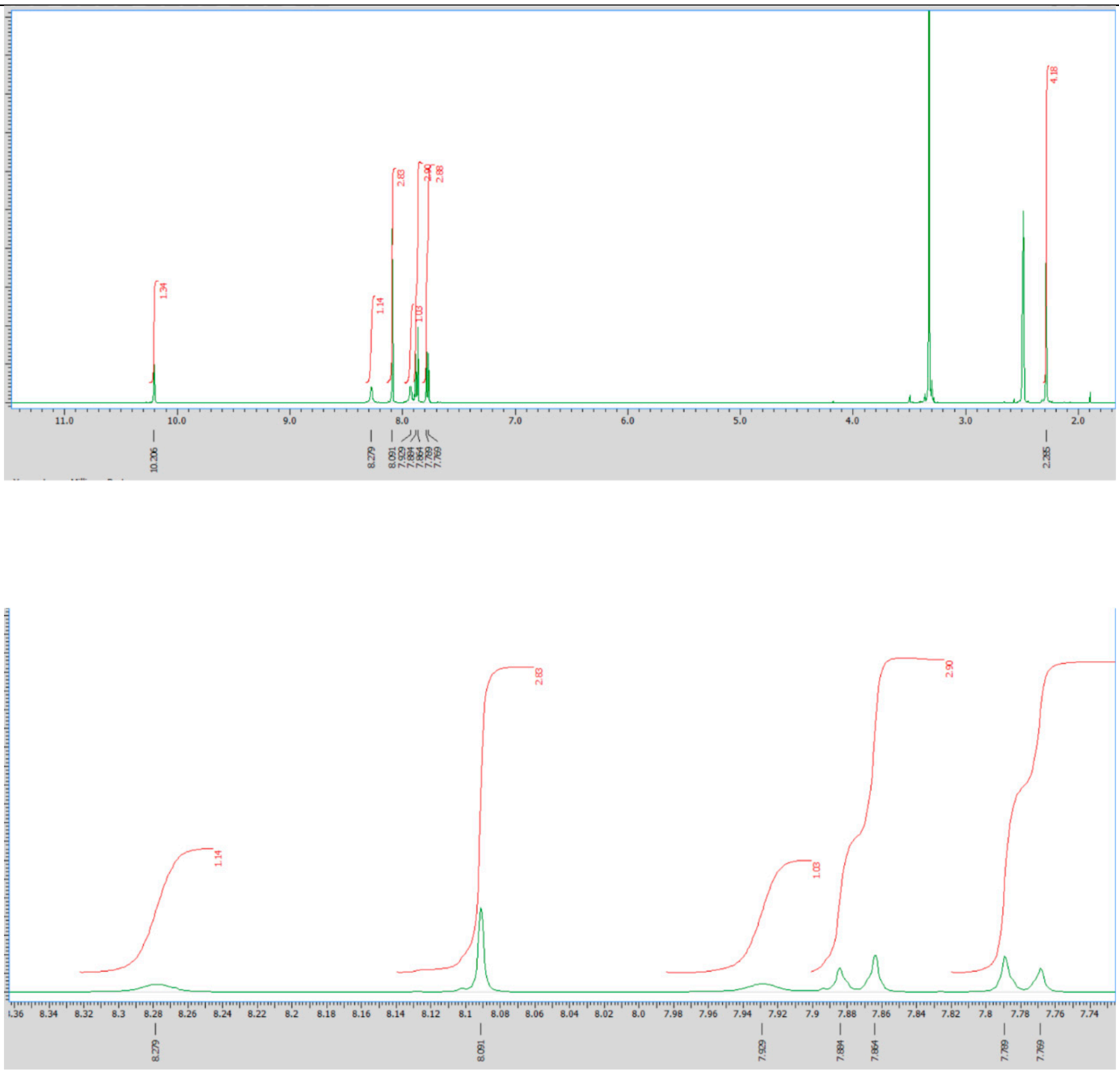
1.34
1.14
2.83
1.03
2.90
2.88
4.18
11.0
10.0
9.0
8.0
7.0
6.0
5.0
4.0
3.0
2.0
10.206
8.279
8.091
7.929
7.884
7.864
7.789
7.769
2.285
8.34
8.32
8.3
8.28
8.26
8.24
8.22
8.2
8.18
8.16
8.14
8.12
8.1
8.08
8.06
8.04
8.02
7.98
7.96
7.94
7.92
7.9
7.88
7.86
7.84
7.82
7.8
7.78
7.76
7.74

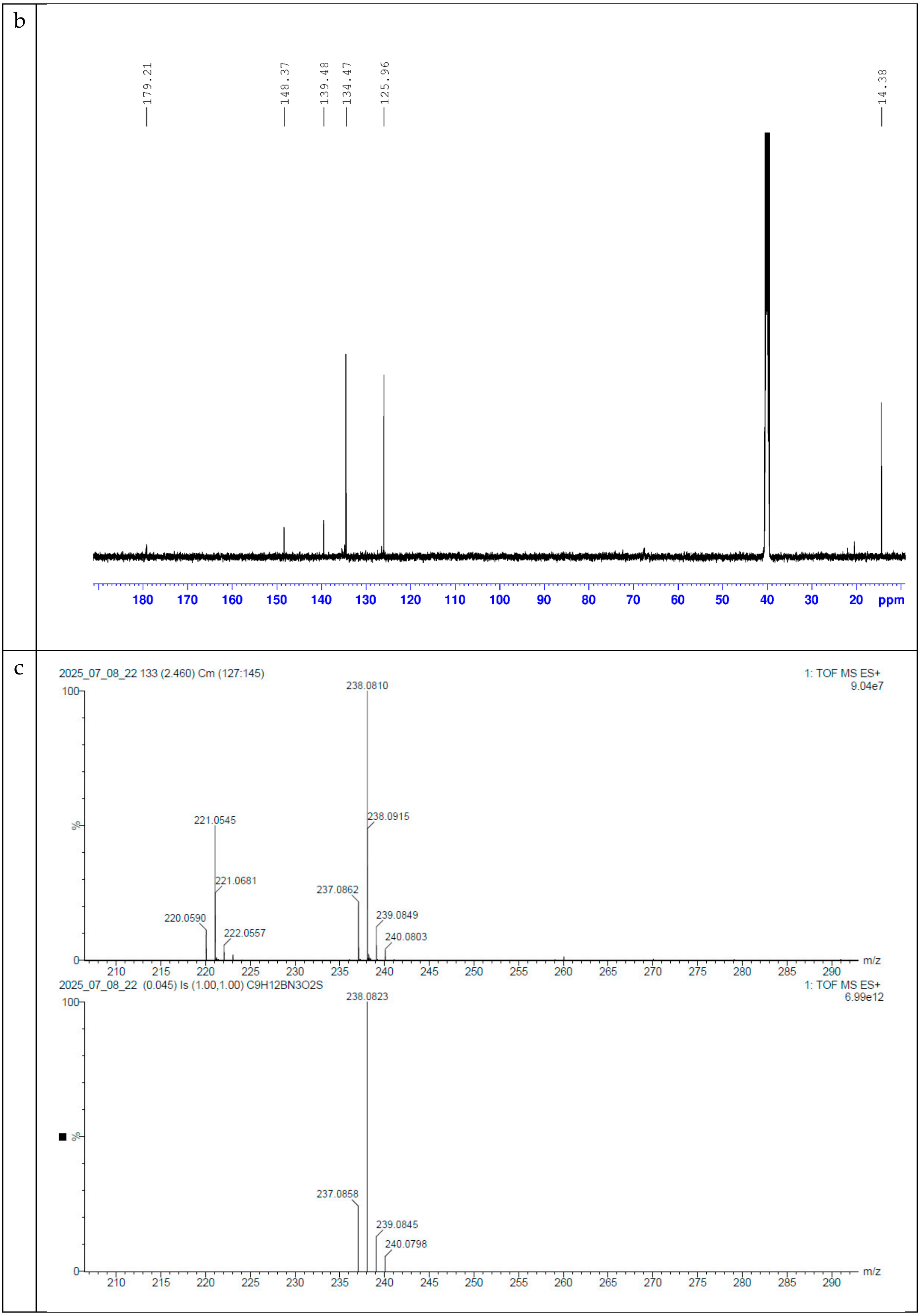

Figure S3. Spectra for compound **6** a) ^{1}H NMR, b) ^{13}C NMR, c) HRMS

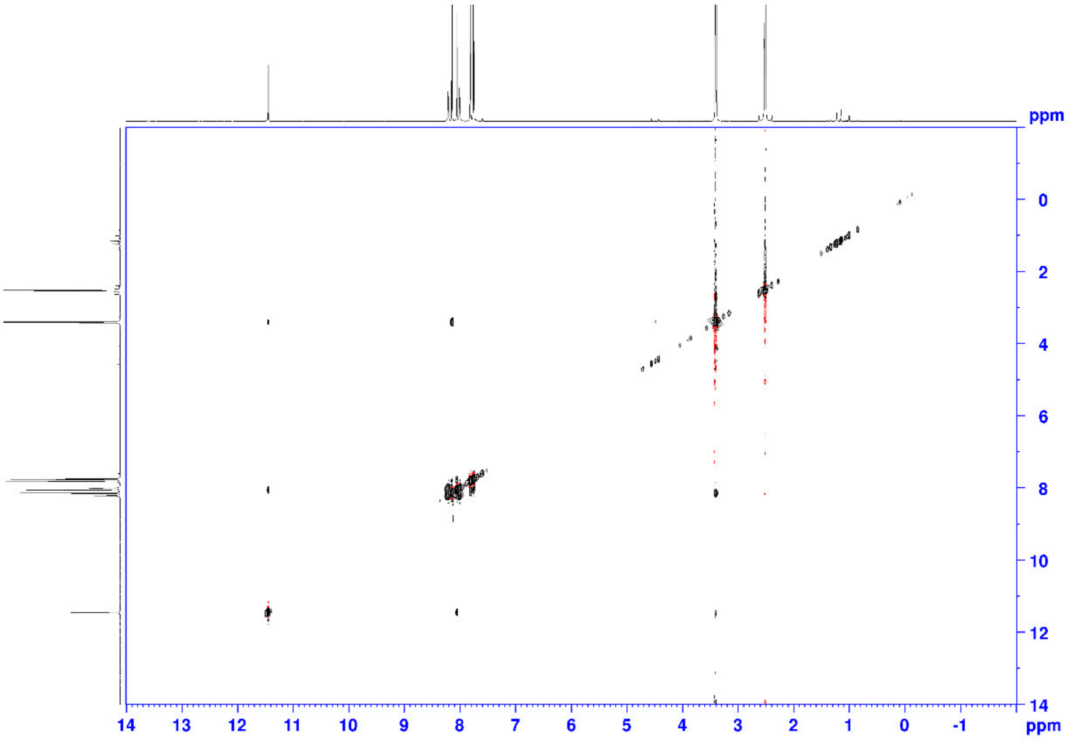

Figure S4. 2D NOESY spectra for compound **3**

Inhibitor activity

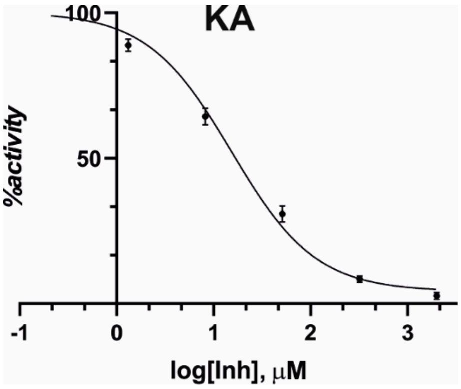

Figure S5. Dose-response curves for the determination of IC_{50} for kojic acid (**KA**)

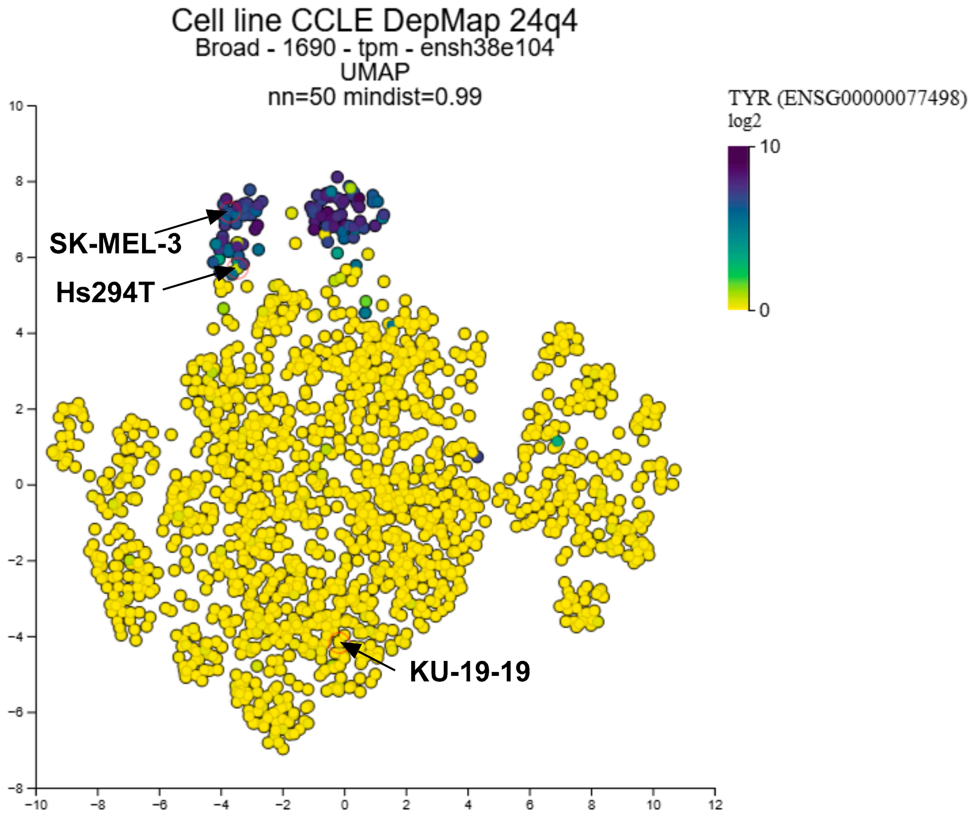

Figure S6. Visualization of the Cancer Cell Line Encyclopedia (CCLE) using Uniform Manifold Approximation and Projection (UMAP). The plot highlights the cell lines chosen for this work, and the color scale corresponds to their respective tyrosinase (TYR) gene expression levels. The analysis was performed using the R2 online platform (r2.amc.nl).

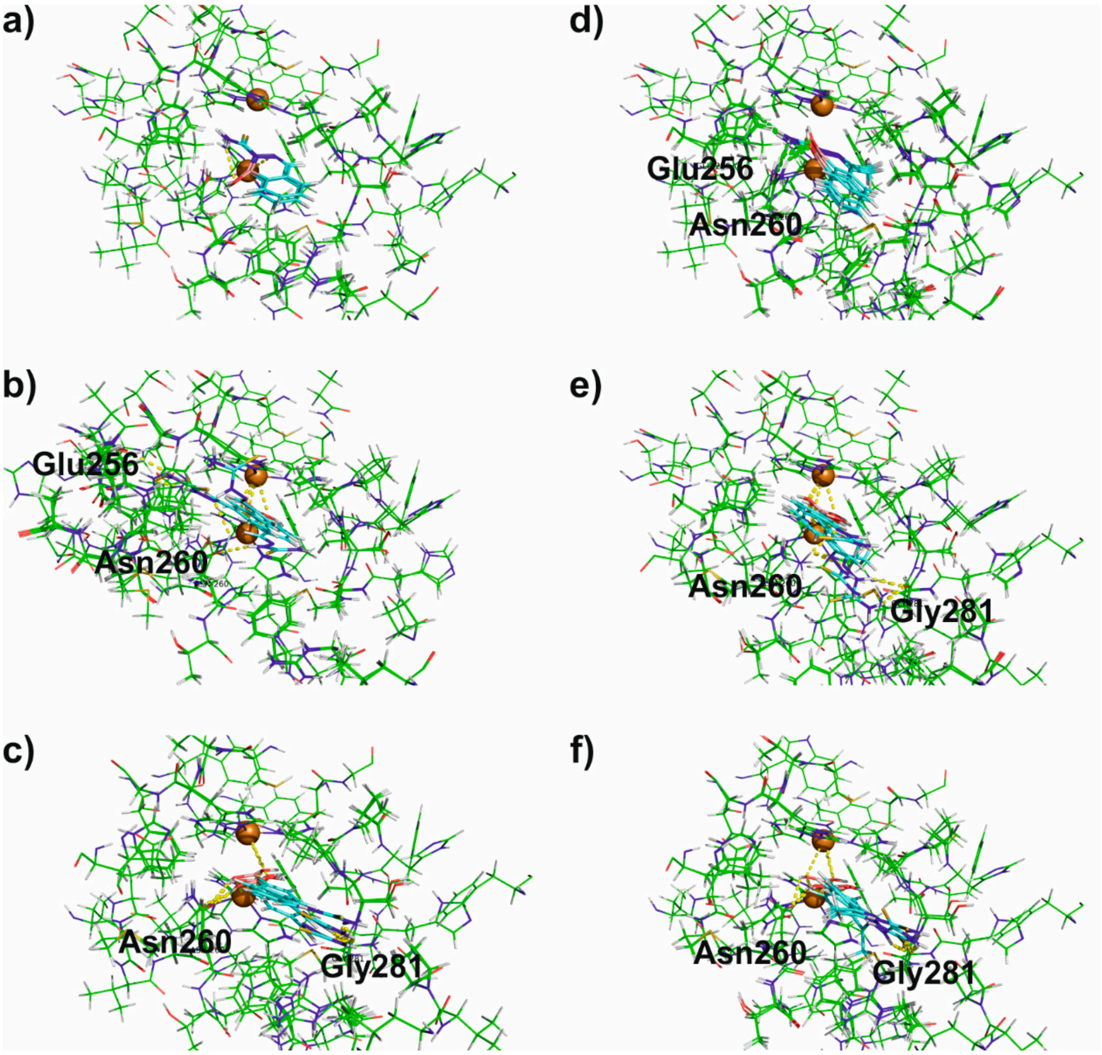

Figure S7. Visualization of alignment of the three best locations for a) **1;** b) **2**; c) **3**; d) **4**; e) **5**; f) **6** in the tyrosine active site. Inhibitors are shown as blue sticks, enzyme residues are shown as green lines, and copper ions are shown as gold spheres. Enzyme's residues involved in the intermolecular H-bond are labeled, and H-bonds are shown as a dashed line.

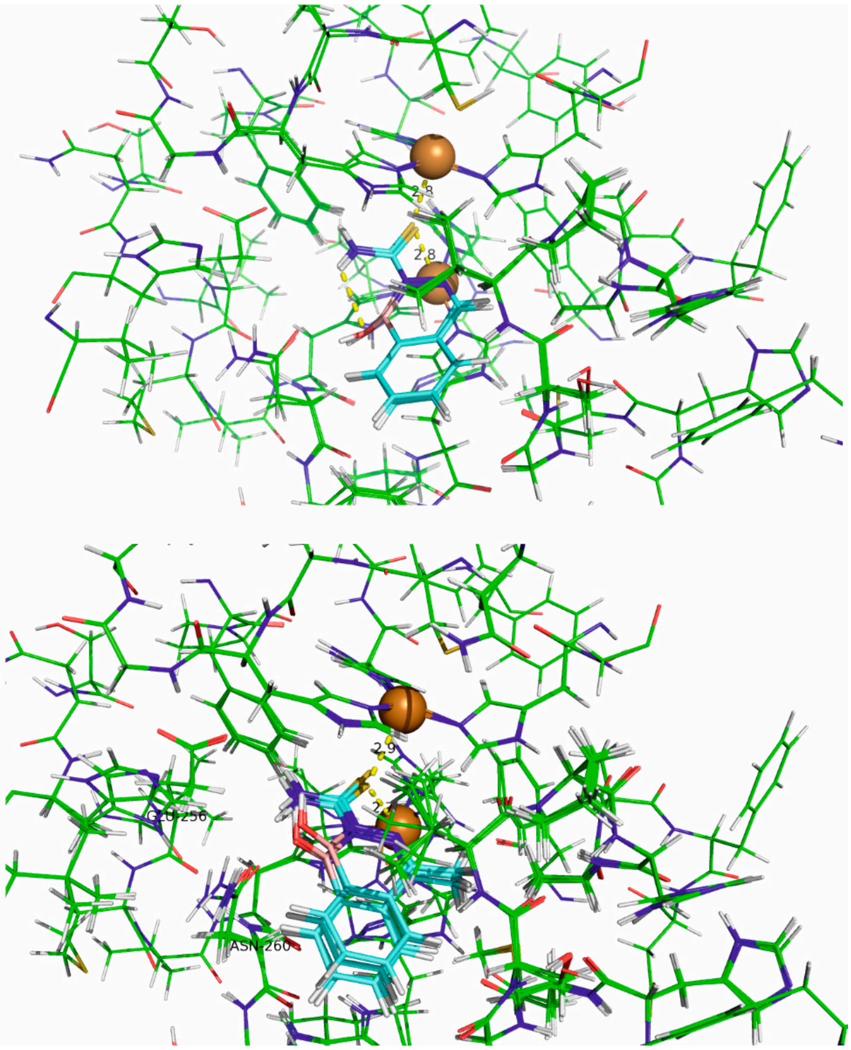

Figure S8. Distances of the thiourea sulfur atom for inhibitors 1 (top) and 4 (bottom) from the cupric ions of tyrosinase. Copper ions are marked as gold spheres.

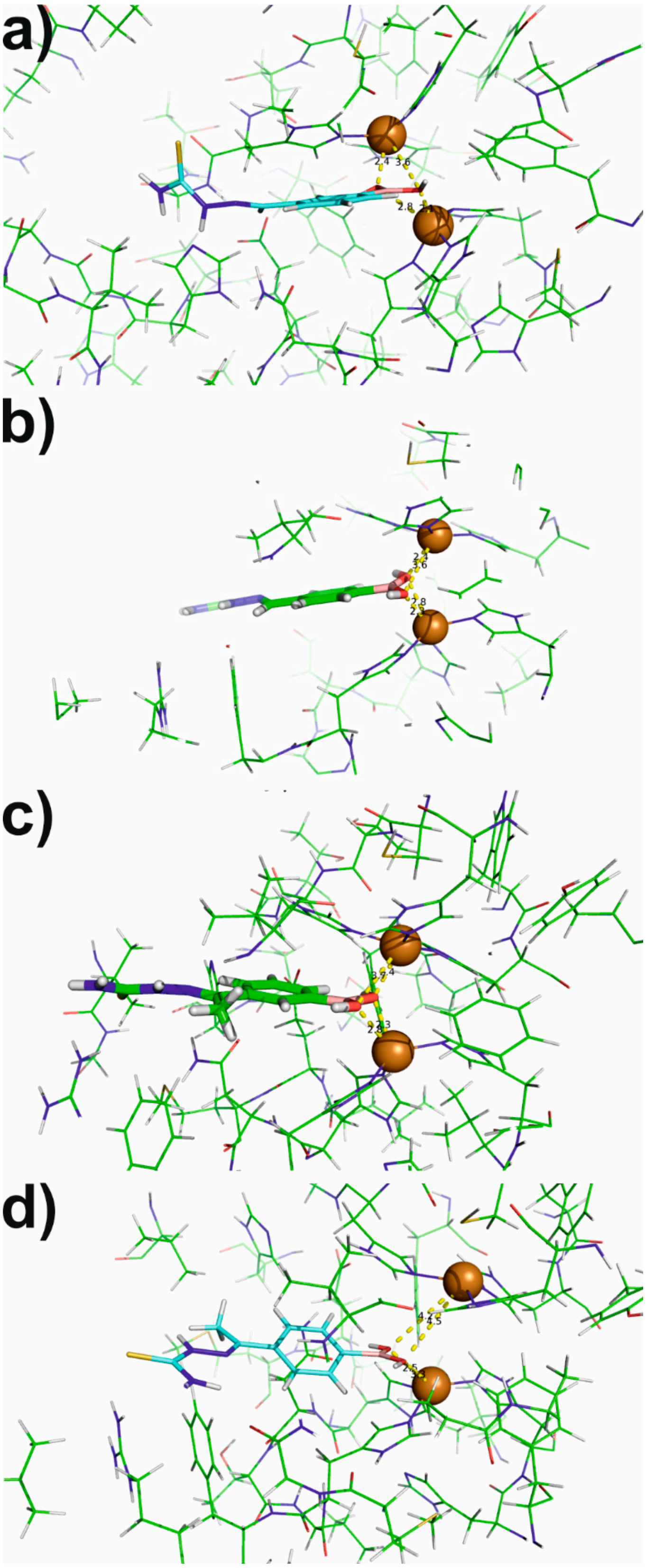

Figure S9. Distances of oxygen atoms of the boron group for inhibitors a) 2; b) 3; c) 5, d) 6 from the cupric ions of tyrosinase. Cupric ions marked as gold spheres

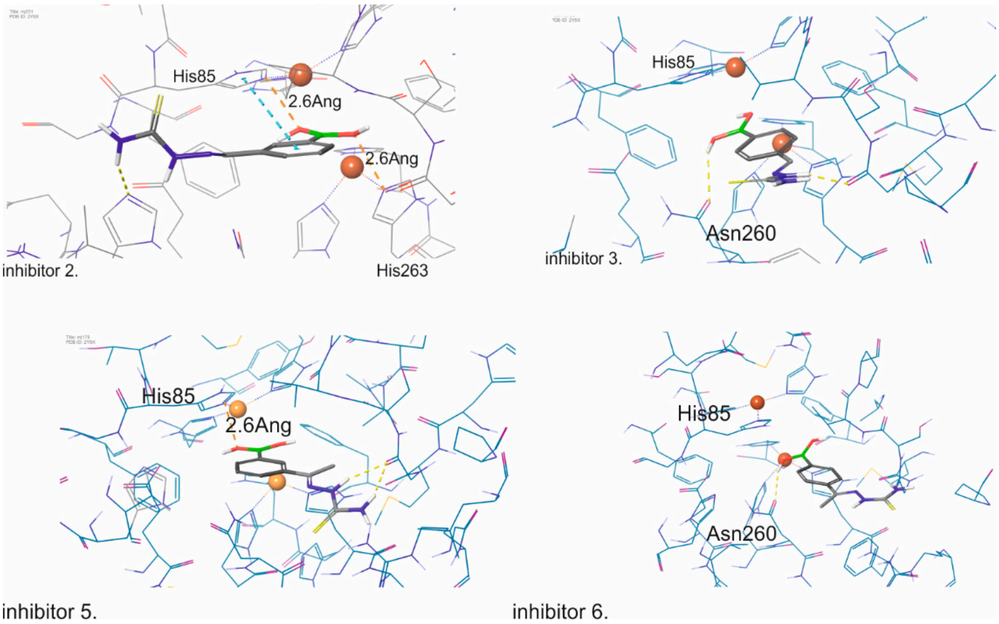

Figure S10. Alignment of residues 2, 3, 5, and 6 in the tyrosinase active site, with marker unfavorable interactions (orange dashed line) between the boronic oxygen and the atom NE2 of the histidine sidechain ring. The yellow dashed lines represent intermolecular hydrogen bonds.
